# Neurodegenerative and psychiatric diseases among families with amyotrophic lateral sclerosis

**DOI:** 10.1212/WNL.0000000000004179

**Published:** 2017-08-08

**Authors:** Elisa Longinetti, Daniela Mariosa, Henrik Larsson, Weimin Ye, Caroline Ingre, Catarina Almqvist, Paul Lichtenstein, Fredrik Piehl, Fang Fang

**Affiliations:** From the Departments of Medical Epidemiology and Biostatistics (E.L., D.M., H.L., W.Y., C.A., P.L., F.F.) and Clinical Neuroscience (C.I., F.P.), Karolinska Institutet, Solna; Department of Medical Sciences (H.L.), Örebro University; and Astrid Lindgren Children's Hospital, Lung and Allergy Unit (C.A.), Karolinska University Hospital, Solna, Sweden.

## Abstract

**Objective:**

To estimate risks of neurodegenerative and psychiatric diseases among patients with amyotrophic lateral sclerosis (ALS) and their families.

**Methods:**

We conducted a register-based nested case-control study during 1990–2013 in Sweden to assess whether patients with ALS had higher risks of other neurodegenerative and psychiatric diseases before diagnosis. We included 3,648 patients with ALS and 36,480 age-, sex-, and county of birth–matched population controls. We further conducted a follow-up study of the cases and controls to assess the risks of other neurodegenerative and psychiatric diseases after ALS diagnosis. To assess the potential contribution of familial factors, we conducted similar studies for the relatives of patients with ALS and their controls.

**Results:**

Individuals with previous neurodegenerative or psychiatric diseases had a 49% increased risk of ALS (odds ratio 1.49, 95% confidence interval 1.35–1.66) compared to individuals without these diseases. After diagnosis, patients with ALS had increased risks of other neurodegenerative or psychiatric diseases (hazard ratio 2.90, 95% confidence interval 2.46–3.43) compared to individuals without ALS. The strongest associations were noted for frontotemporal dementia, Parkinson disease, other dementia, Alzheimer disease, neurotic disorders, depression, stress-related disorders, and drug abuse/dependence. First-degree relatives of patients with ALS had higher risk of neurodegenerative diseases, whereas only children of patients with ALS had higher risk of psychiatric disorders, compared to relatives of the controls.

**Conclusions:**

Familial aggregation of ALS and other neurodegenerative diseases implies a shared etiopathogenesis among all neurodegenerative diseases. The increased risk of psychiatric disorders among patients with ALS and their children might be attributable to nonmotor symptoms of ALS and severe stress response toward the diagnosis.

Amyotrophic lateral sclerosis (ALS) overlaps clinically and pathologically with other neurodegenerative diseases.^1–5^ Family members of patients with ALS have also been reported to have increased risks of dementia and Parkinson disease (PD),^6,7^ further supporting the hypothesis of a shared etiopathogenesis between ALS and other neurodegenerative diseases.^6,8,9^ Increased risk of psychiatric disorders has been suggested among patients with ALS in some but not all studies^10–12^ and little is known for the risk of psychiatric disorders among families of patients with ALS.^6^ We performed a nationwide register-based study in Sweden to estimate the risk of neurodegenerative and psychiatric diseases among patients with ALS and their family members.

## METHODS

### Study base.

The Swedish Multi-Generation Register includes information on familial links for all individuals born in Sweden since 1932.^13^ We defined our study population as all individuals included in this register who were born in Sweden during 1932–2013 (n = 8,575,515). Using the unique personal identification numbers assigned to all Swedish residents,^14^ we followed the study population from January 1, 1990, or date of birth, whichever came later, until date of ALS diagnosis, death, emigration out of Sweden, or December 31, 2013, whichever came first, through cross-linkages to the Swedish Patient Register, Causes of Death Register, and Migration Register. The Patient Register collects data on hospital discharge records in Sweden since 1964 and has a nationwide coverage since 1987.^14^ Since 2001, it also collects data on hospital-based outpatient specialist care. Diagnoses from each hospital visit are classified according to the Swedish revisions of the International Classification of Disease (ICD) codes (ICD-7 before 1969, ICD-8 during 1969–1986, ICD-9 during 1987–1996, and ICD-10 from 1997). We identified all newly diagnosed ALS cases during follow-up through the Patient Register, indicated by a hospital visit concerning ALS, and defined the first hospital visit as ALS diagnosis date. We ascertained date of death from the Causes of Death Register and date of first emigration out of Sweden from the Migration Register. We excluded individuals who had been diagnosed with ALS (n = 662), died (n = 120,612), or emigrated out of Sweden (n = 186,670) before the beginning of follow-up, leaving 8,269,319 (96%) participants in the study base.

### Nested case-control study I.

We conducted a nested case-control study within the above study base to assess the association of previous neurodegenerative and psychiatric diseases with the subsequent ALS risk. We defined cases as individuals diagnosed with ALS during follow-up (ICD-9 code 335C, ICD-10 code G12.2; n = 3,648). For each index case, we randomly selected 10 controls from the study base, by incidence density sampling, and individually matched the controls to the cases by year and month of birth, sex, and county of birth (n = 36,480). Eligible controls had to be alive, living in Sweden, and ALS-free, at the time of the diagnosis of the index case.

### Nested case-control study II.

To investigate whether relatives of patients with ALS had increased risk of neurodegenerative and psychiatric diseases before the diagnosis of the proband ALS patient, we conducted a second nested case-control study, including relatives of the index patients with ALS and their matched controls. We identified parents, full siblings, half-siblings, and children of the index cases and controls from the Multi-Generation Register and used them as cases and controls for the nested case-control study II. We used the index dates of the index cases and controls as the index dates for the respective relatives. We excluded from the analyses relatives who had died or emigrated out of Sweden before the index date.

### Follow-up studies.

To examine the relative risks of neurodegenerative and psychiatric diseases after ALS diagnosis, we prospectively followed the above nested case-control studies from the index date. In these analyses, we included only individuals without any neurodegenerative or psychiatric diseases diagnosed prior to the index dates, leading to 3,169 ALS cases and 33,110 controls in the follow-up study of nested case-control study I, and 13,313 relatives of patients with ALS and 130,321 relatives of the index controls in the follow-up study of the nested case-control study II. We followed all individuals from the index date to the date of first diagnosis of neurodegenerative or psychiatric diseases, death, emigration out of Sweden, or December 31, 2013, whichever came first.

### Ascertainment of neurodegenerative and psychiatric diseases.

Neurodegenerative diseases examined in this study included frontotemporal dementia (FTD), Alzheimer disease (AD), other or unspecific dementia, and PD, because we had previously reported risk of ALS among relatives of patients with ALS.^15^ Psychiatric disorders examined in this study included schizophrenia, bipolar disorder, depression, neurotic disorders, stress-related disorders, alcohol abuse/dependence, and drug abuse/dependence. We defined a diagnosis of these diseases through a hospital visit concerning the specific disease as recorded in the Patient Register and used the date of first hospital visit as the diagnosis date. Because the Patient Register achieved good coverage on psychiatric diagnoses in 1973,^16^ we ascertained the diagnoses of neurodegenerative and psychiatric diseases from 1973 until the index date for the nested case-control studies and from the index date until the end of follow-up for the follow-up studies. A list of the corresponding ICD codes is provided in table e-1 at Neurology.org.

### Statistical analysis.

In the nested-case control studies, we estimated odds ratios (ORs) of ALS (or becoming a relative of an ALS patient) and corresponding 95% confidence intervals (CIs) using conditional logistic regression as measures of the associations between previous neurodegenerative or psychiatric diseases and the subsequent ALS risk. Because cases and controls were individually matched by year and month of birth, sex, and county of birth in the nested case-control study I, these variables were automatically adjusted for in the analyses. In the nested case-control study II, we adjusted all models for year and month of birth, sex, and county of birth of the relatives as well as of the proband individuals.

In the follow-up studies, we fitted Cox proportional hazard regression to derive hazard ratios (HRs) for the future risk of neurodegenerative and psychiatric diseases, comparing patients with ALS to their controls and the relatives of patients with ALS to the relatives of the index controls. We used attained age as the underlying time scale and further adjusted all models for sex and county of birth. We tested the assumption of proportional hazards using Schoenfeld residuals.

In addition to the overall analyses, we separately analyzed specific time windows in the nested case-control studies and the follow-up studies (≥6 years, 2–5 years, or 0–1 year before and after the index date). The period ≤1 year before ALS diagnosis might be representative of a time period of symptoms onset and clinical diagnostic workup.

To assess the potential influence of age and sex on the studied associations, we separately analyzed men and women and individuals at age ≤55 and at age ≥56. Because familial ALS cases might have different association with other neurodegenerative diseases, compared to sporadic ALS cases, we separately analyzed ALS cases with a family history. By crosslinking the nested case-control study I to the Multi-Generation Register, we identified grandparents, parents, uncles/aunts, full and half siblings, children, nephews/nieces, and grandchildren of patients with ALS and their corresponding controls. We then linked these relatives to the Patient Register to obtain ALS diagnosis among them and defined a family history of ALS as having at least one of these relatives diagnosed with ALS until the end of 2013.

To investigate if misdiagnosis of neurodegenerative diseases might explain some of the associations between other neurodegenerative diseases and ALS, we conducted an additional analysis by restricting the definition of ALS and other neurodegenerative diseases to patients with at least 2 hospital visits concerning respective diseases. Given the established ALS/FTD overlap,^17^ we additionally assessed the risk of other neurodegenerative diseases among patients with ALS after excluding FTD from the definition of neurodegenerative diseases. Finally, as depression, neurotic disorders, and stress-related disorders might represent collectively the psychological burden of ALS symptoms and diagnosis on patients with ALS and their families, we analyzed the risk of having depression, neurotic disorders, or stress-related disorders before and after ALS diagnosis.

We considered statistically significant associations with a 2-sided *p* value ≤0.05. We performed analyses using Stata software, version 14 (StataCorp, College Station, TX).

### Standard protocol approvals, registrations, and patient consents.

The Regional Ethical Review Board in Stockholm, Sweden, approved this study.

## RESULTS

Table 1 shows the sex and age distributions of patients with ALS, their controls, and the relatives of both groups. The mean age at diagnosis of patients with ALS was 60 years (SD 11.30).

**Table 1:**
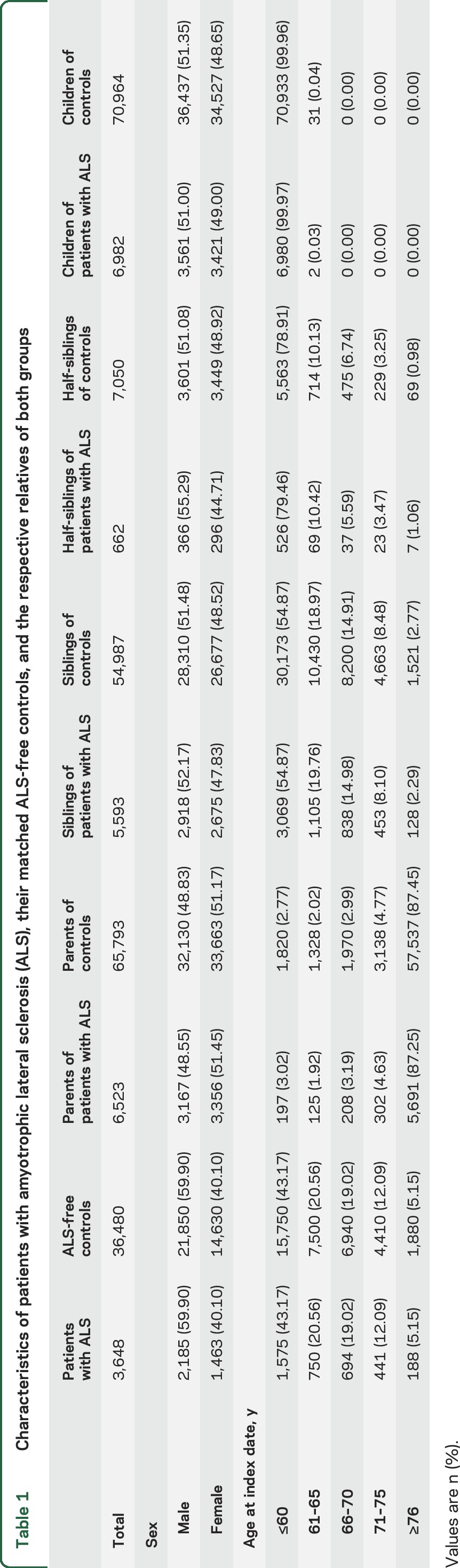
Characteristics of patients with amyotrophic lateral sclerosis (ALS), their matched ALS-free controls, and the respective relatives of both groups

Both before and after the index date, we found higher risks for all neurodegenerative diseases studied and for depression, neurotic disorders, and drug abuse/dependence among patients with ALS, compared to controls (table 2). The associations were strongest for FTD, followed by PD, other or unspecific dementia, and AD. Because the proportional hazards assumption was violated after age 68 in the analyses of any neurodegenerative disease and other or unspecific dementia, we restricted these analyses to attained age <69 years and found largely similar results (table e-2).

**Table 2:**
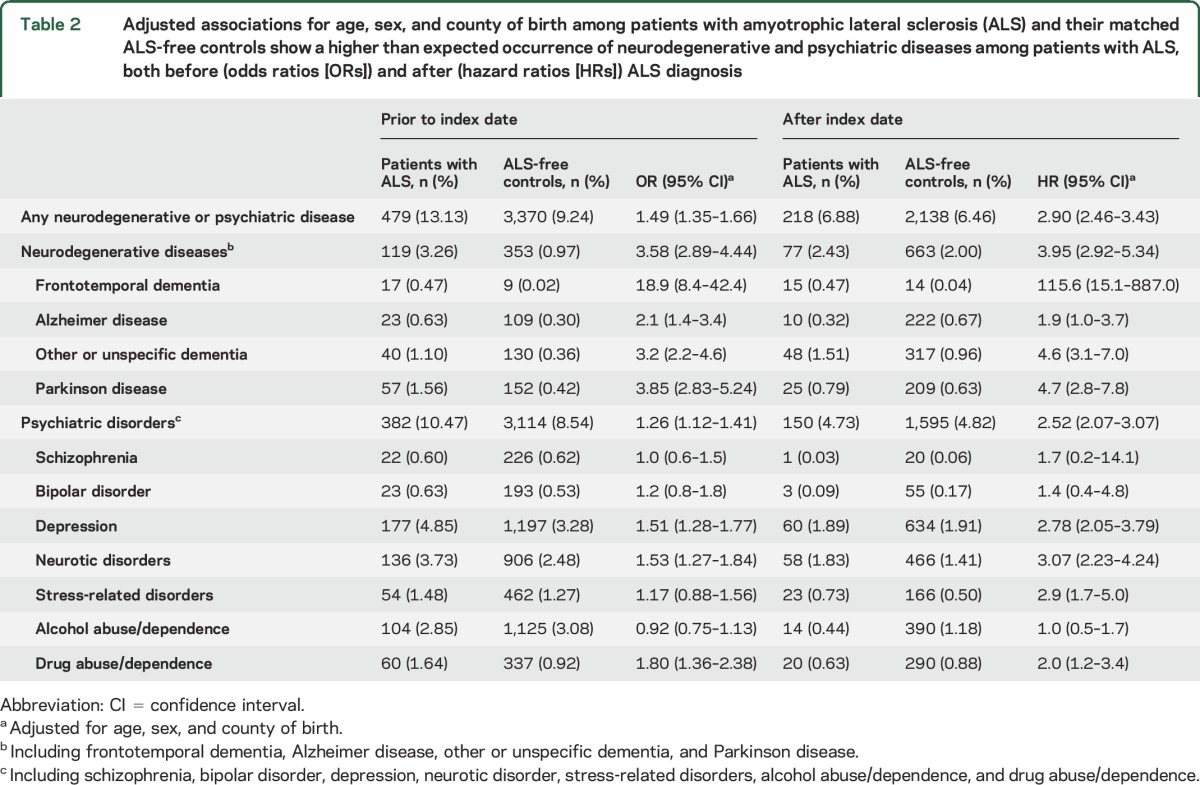
Adjusted associations for age, sex, and county of birth among patients with amyotrophic lateral sclerosis (ALS) and their matched ALS-free controls show a higher than expected occurrence of neurodegenerative and psychiatric diseases among patients with ALS, both before (odds ratios [ORs]) and after (hazard ratios [HRs]) ALS diagnosis

Overall, parents, siblings, and children of patients with ALS had higher risk of neurodegenerative diseases, both before and after the index date, compared to relatives of ALS-free individuals, although the associations were only statistically significant for siblings (table 3). Children of patients with ALS had higher risks of psychiatric disorders both before and after the index date, compared to children of the controls.

**Table 3:**
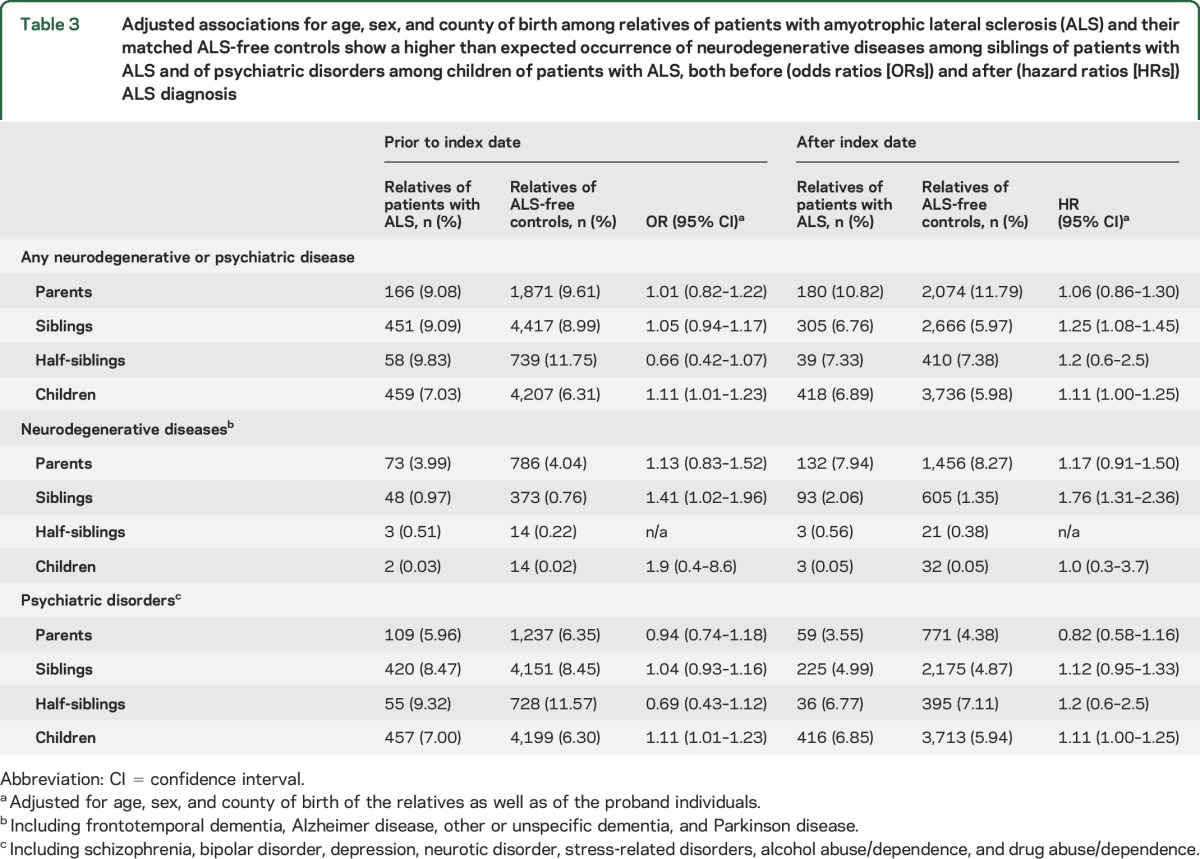
Adjusted associations for age, sex, and county of birth among relatives of patients with amyotrophic lateral sclerosis (ALS) and their matched ALS-free controls show a higher than expected occurrence of neurodegenerative diseases among siblings of patients with ALS and of psychiatric disorders among children of patients with ALS, both before (odds ratios [ORs]) and after (hazard ratios [HRs]) ALS diagnosis

The associations of FTD, AD, other or unspecific dementia, PD, depression, neurotic disorders, and drug abuse/dependence with the subsequent ALS risk appeared to be strongest during the year before ALS diagnosis, although we also noted positive associations during 2–5 years before diagnosis (table 4). We observed further positive associations for schizophrenia and stress-related disorders during the year before ALS diagnosis. We noted similar patterns for the associations of ALS with the subsequent risks of neurodegenerative or psychiatric diseases, with the strongest association during the first year after diagnosis, followed by 2–5 years after diagnosis (table 4). Patients with ALS had an increased risk of stress-related disorders after diagnosis, especially during the first year. A clear temporal pattern was not identified for the analyses of the relatives (table e-3).

**Table 4:**
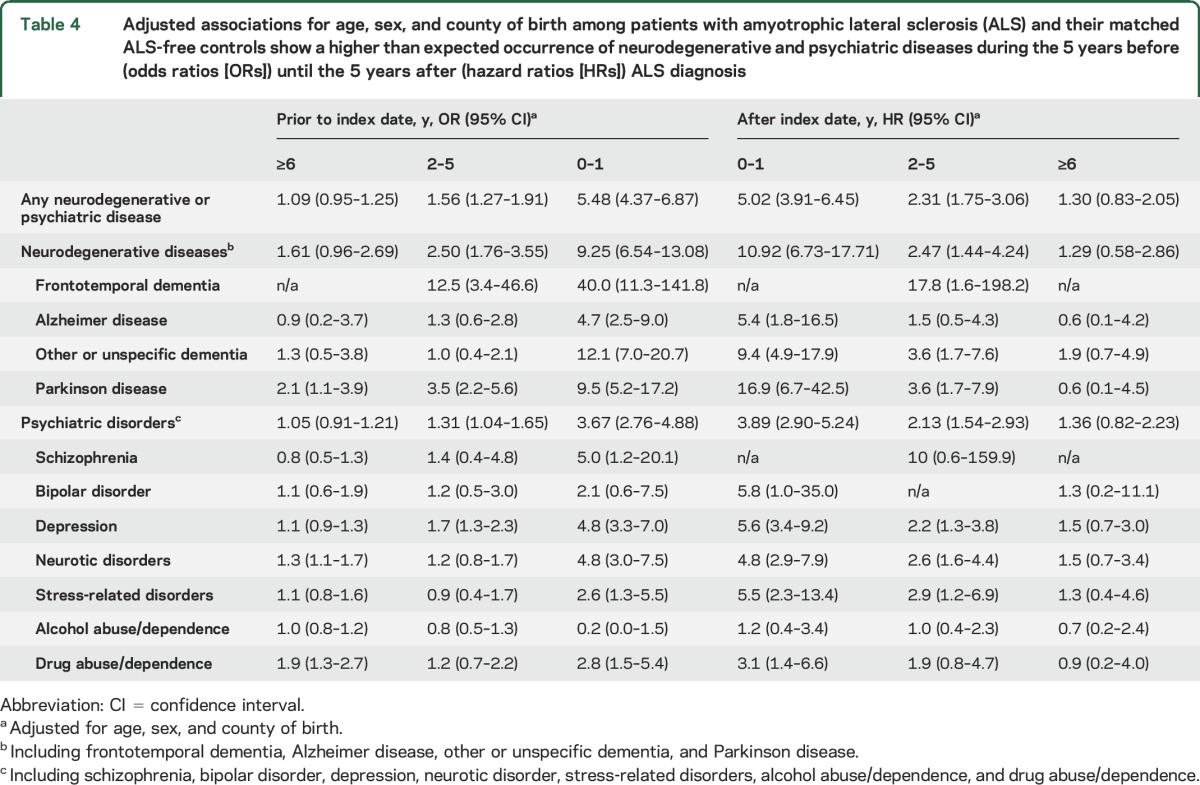
Adjusted associations for age, sex, and county of birth among patients with amyotrophic lateral sclerosis (ALS) and their matched ALS-free controls show a higher than expected occurrence of neurodegenerative and psychiatric diseases during the 5 years before (odds ratios [ORs]) until the 5 years after (hazard ratios [HRs]) ALS diagnosis

All results of the main analyses appeared comparable between males and females (tables e-4 and e-5) and between individuals younger or older than 55 years (tables e-6 and e-7). We identified a total of 173 patients with ALS with a family history of ALS (3.9%); separate analysis of these familial ALS cases generally provided similar results as in the main analyses (table e-8).

Among the 3,648 patients with ALS in our study, 3,048 had at least 2 hospital records concerning ALS, and among the 600 cases with only one record, 246 had ALS as a cause of death in the Causes of Death Register. Restricting the definition of ALS and other neurodegenerative diseases to patients with at least 2 hospital visits concerning respective diseases slightly attenuated the associations between other neurodegenerative diseases and ALS both prior to (OR 2.02, 95% CI 1.48–2.77) and after (HR 3.27, 95% CI 2.14–5.00) ALS diagnosis. Excluding FTD from the definition of neurodegenerative diseases attenuated slightly the associations of ALS with other neurodegenerative diseases in the overall analyses (prior to index date, OR 3.34, 95% CI 2.68–4.16; after index date, HR 3.88, 95% CI 2.87–5.26) and the temporal pattern analyses (table e-9).

The risk of having depression, neurotic disorders, or stress-related disorders was significantly higher among patients with ALS, compared to controls, both before (OR 1.46, 95% CI 1.28–1.66) and after (HR 3.13, 95% CI 2.50–3.92) ALS diagnosis. The risk increase peaked during the year before (OR 4.87, 95% CI 3.58–6.62) and the year after (HR 5.52, 95% CI 3.93–7.76) ALS diagnosis.

## DISCUSSION

Using a nationwide population-based study sample, we found that patients with ALS had higher risks of neurodegenerative and psychiatric diseases, both before and after diagnosis. Parents, siblings, and children of patients with ALS tended to have increased risk of neurodegenerative diseases, whereas only children of patients with ALS had increased risk of psychiatric disorders.

Although previous studies reported increased risks of dementia and PD after ALS diagnosis,^3,4^ our study is the first to demonstrate the temporal pattern of the increased risks from years before until years after ALS diagnosis. We further showed that parents, siblings, and children of patients with ALS tended also to have increased risks of other neurodegenerative diseases, corroborating findings of a recent study in Ireland.^6^ Our results lend further support to the hypothesis that shared etiologies or disease mechanisms might underlie different neurodegenerative diseases.^6,8,9^ Such mechanisms might include shared genetic risk factors,^1,18^ leading for example to accumulation of protein aggregates in the brain, a common pathologic finding from different neurodegenerative diseases.^19^ Nongenetic risk factors such as exposure to agrochemicals and previous head trauma have also been linked to different neurodegenerative diseases.^20^

The stronger associations with neurodegenerative diseases noted during the 5 years before and after ALS diagnosis were not reported previously and might be due to different reasons. Misdiagnosis between ALS and other neurodegenerative diseases could contribute partially to the increased risk of other neurodegenerative diseases among patients with ALS. The diagnosis of ALS in the Patient Register appears to have high accuracy because a validation study of 280 patients in Stockholm showed a positive predictive value of 91% for medical records–based ALS diagnosis.^21^ Restricting the definition of ALS and other neurodegenerative diseases to patients with at least 2 hospital visits concerning respective diseases attenuated slightly, but did not diminish the results, arguing against misdiagnosis as the pure explanation for the observed associations. Furthermore, patients with ALS might have been more closely surveyed and more likely to receive a diagnosis of another neurodegenerative disease compared to ALS-free individuals, leading to a higher than expected risk of other neurodegenerative diseases. It is also possible that some symptoms of other neurodegenerative diseases become underdetected because of the predominant ALS symptoms.

We found an increased risk of psychiatric disorders among patients with ALS both before and after diagnosis. The increased risk of depression is in line with previous reports.^11,12^ The increased risks of neurotic disorders and stress-related disorders are not surprising, because depression, neurotic disorders, and stress-related disorders are highly correlated clinically.^22^

The stronger associations with psychiatric disorders noted during the 5 years before and after ALS diagnosis might be due to both nonmotor symptoms of ALS and severe stress response toward these symptoms and the final diagnosis. Nonmotor symptoms of ALS including cognitive impairment are increasingly recognized^23^ and may mimic psychiatric symptoms.^24^ The increased risk of depression might partially represent increased prevalence of cognitive impairment among patients with ALS. The increased risks of depression, neurotic disorders, and stress-related disorders, peaking during the year before and after ALS diagnosis, might on the other hand collectively suggest a reactive nature of these psychiatric disorders, potentially due to the emotional burden of ALS symptoms and diagnosis.

In line with a previous study that identified an association of schizophrenia with subsequent ALS,^11^ we noted an increased risk of schizophrenia during the 5 years before ALS diagnosis, although the association was only statistically significant during the year before diagnosis. A recent genome-wide association study also suggested a genetic overlap between ALS and schizophrenia.^25^

In contrast to previous studies,^26–29^ we did not find an association between alcohol abuse/dependence and a lower ALS risk. Lack of adjustment for smoking and total energy intake in the present study might partially explain these conflicting results. In accord with the previously suggested association between use of opioids and ALS,^30^ our study reports a higher risk of drug abuse or dependence (including medicines, cocaine, caffeine, opioids, and cannabis) among patients with ALS, both before and after ALS diagnosis. While drug abuse/dependence might be partially secondary to depression and stress-related disorders,^31–33^ these associations diminished but did not disappear after excluding individuals with concurrent drug abuse/dependence, depression, or stress-related disorders (data not shown).

We observed an increased risk of psychiatric disorders among children, but not siblings or parents, of patients with ALS. The vast majority of children of patients with ALS who received a psychiatric diagnosis (n = 742, 81%) received a diagnosis of depression, neurotic disorders, or stress-related disorders, suggesting that psychological distress was likely the primary reason for such increased risk. This is possibly explained by the fact that children are more involved in caring for patients with ALS compared to other relatives.^34^

Main strengths of our study are the large sample size and the population-based design. The long-term study period and the complete follow-up, the prospectively collected information, as well as the ability to objectively identify family members and their disease history, represent other main strengths.

We lacked information on the genetic and clinical characteristics of patients with ALS, and were therefore unable to separately analyze different subtypes of ALS. Although the completeness of ALS diagnosis is presumably high in the Swedish Patient Register because all patients with ALS are diagnosed by a specialist, we might have underestimated the prevalence of some neurodegenerative and psychiatric diseases because health care provided by general practitioners is not included in the register. The 1% prevalence of FTD among the patients with ALS might reflect a lack of FTD detection.^35^ Some of the patients with FTD might have been misclassified as other dementia, partially accounting for the increased risk of other dementia among patients with ALS. Because the vast majority of children of patients with ALS were younger than 60 years, risk of neurodegenerative diseases at older ages of children needs to be further assessed. Although the clear temporal pattern before and after ALS diagnosis argues against confounding as an important explanation for the noted associations, residual confounding remains a possibility. Finally, whether or not these findings are generalizable to other populations needs to be tested in further studies.

We found that patients with ALS and their first-degree relatives had increased risks of neurodegenerative diseases before and after diagnosis, lending further support to a common etiopathogenesis for different neurodegenerative diseases. The increased risk of psychiatric disorders among patients with ALS and their children might be attributable to both the nonmotor symptoms of ALS and severe stress response to the progressive symptoms and diagnosis of a fatal disease.

## Supplementary Material

Data Supplement

## GLOSSARY

AD: Alzheimer disease
ALS: amyotrophic lateral sclerosis
CI: confidence interval
FTD: frontotemporal dementia
HR: hazard ratio
ICD: International Classification of Disease
OR: odds ratio
PD: Parkinson disease

## References

[1] Andersen PM, Al-Chalabi A. Clinical genetics of amyotrophic lateral sclerosis: what do we really know? Nat Rev Neurol 2011;7:603–615. 21989245 10.1038/nrneurol.2011.150

[2] Cavaleri F. Review of amyotrophic lateral sclerosis, Parkinson's and Alzheimer's diseases helps further define pathology of the novel paradigm for Alzheimer's with heavy metals as primary disease cause. Med Hypoth 2015;85:779–790. 10.1016/j.mehy.2015.10.009 26604027

[3] Li X, Sundquist J, Sundquist K. Subsequent risks of Parkinson disease in patients with autoimmune and related disorders: a nationwide epidemiological study from Sweden. Neurodegener Dis 2012;10:277–284. 22205172 10.1159/000333222

[4] Korner S, Kollewe K, Ilsemann J, et al. Prevalence and prognostic impact of comorbidities in amyotrophic lateral sclerosis. Eur J Neurol 2013;20:647–654. 23094606 10.1111/ene.12015

[5] Bradshaw WJ, Rehman S, Pham TT, et al. Structural insights into human angiogenin variants implicated in Parkinson's disease and amyotrophic lateral sclerosis. Scientific Rep 2017;7:41996. 10.1038/srep41996 PMC5296752 28176817

[6] Byrne S, Heverin M, Elamin M, et al. Aggregation of neurologic and neuropsychiatric disease in amyotrophic lateral sclerosis kindreds: a population-based case-control cohort study of familial and sporadic amyotrophic lateral sclerosis. Ann Neurol 2013;74:699–708. 23836460 10.1002/ana.23969

[7] Bryan L, Kaye W, Antao V, Mehta P, Muravov O, Horton DK. Preliminary results of national amyotrophic lateral sclerosis (ALS) Registry risk factor Survey data. PLoS One 2016;11:e0153683. 27124833 10.1371/journal.pone.0153683 PMC4849726

[8] Coppede F, Mancuso M, Siciliano G, Migliore L, Murri L. Genes and the environment in neurodegeneration. Biosci Rep 2006;26:341–367. 17029001 10.1007/s10540-006-9028-6

[9] Fallis BA, Hardiman O. Aggregation of neurodegenerative disease in ALS kindreds. Amyotroph Lateral Scler 2009;10:95–98. 18608094 10.1080/17482960802209664

[10] Seelen M, van Doormaal PC, Visser A, et al. Prior medical conditions and the risk of amyotrophic lateral sclerosis. J Neurol 2014;261:1949–1956. 25059395 10.1007/s00415-014-7445-1

[11] Turner MR, Goldacre R, Talbot K, Goldacre MJ. Psychiatric disorders prior to amyotrophic lateral sclerosis. Ann Neurol 2016;80:935–938. 27761925 10.1002/ana.24801 PMC5215396

[12] Roos E, Mariosa D, Ingre C, et al. Depression in amyotrophic lateral sclerosis. Neurology 2016;86:2271–2277. 27164661 10.1212/WNL.0000000000002671 PMC4909561

[13] Ekbom A. The Swedish Multi-generation Register. Methods Mol Biol 2011;675:215–220. 20949391 10.1007/978-1-59745-423-0_10

[14] Ludvigsson JF, Otterblad-Olausson P, Pettersson BU, Ekbom A. The Swedish personal identity number: possibilities and pitfalls in healthcare and medical research. Eur J Epidemiol 2009;24:659–667. 19504049 10.1007/s10654-009-9350-y PMC2773709

[15] Fang F, Kamel F, Lichtenstein P, et al. Familial aggregation of amyotrophic lateral sclerosis. Ann Neurol 2009;66:94–99. 19670447 10.1002/ana.21580 PMC3609703

[16] Ludvigsson JF, Andersson E, Ekbom A, et al. External review and validation of the Swedish national inpatient register. BMC Public Health 2011;11:450. 21658213 10.1186/1471-2458-11-450 PMC3142234

[17] Phukan J, Pender NP, Hardiman O. Cognitive impairment in amyotrophic lateral sclerosis. Lancet Neurol 2007;6:994–1003. 17945153 10.1016/S1474-4422(07)70265-X

[18] Ahmad K, Baig MH, Mushtaq G, Kamal MA, Greig NH, Choi I. Commonalities in biological pathways, genetics, and cellular mechanism between Alzheimer disease and other neurodegenerative diseases: an in silico-updated overview. Curr Alzheimer Res Epub 2017 Feb 3. 10.2174/1567205014666170203141151 PMC5878041 28164765

[19] Khanam H, Ali A, Asif M, Shamsuzzaman. Neurodegenerative diseases linked to misfolded proteins and their therapeutic approaches: a review. Eur J Med Chem 2016;124:1121–1141. 27597727 10.1016/j.ejmech.2016.08.006

[20] de Pedro-Cuesta J, Martinez-Martin P, Rabano A, et al. Drivers: a biologically contextualized, cross-inferential view of the epidemiology of neurodegenerative disorders. J Alzheimers Dis 2016;51:1003–1022. 26923014 10.3233/JAD-150884 PMC4927850

[21] Mariosa D, Hammar N, Malmström H, et al. Blood biomarkers of carbohydrate, lipid and apolipoprotein metabolism and risk of amyotrophic lateral sclerosis: a more than 20 years follow-up of the Swedish AMORIS cohort. 27th International Symposium on ALS/MND. Dublin, Ireland: Amyotrophic Lateral Sclerosis and Frontotemporal Degeneration, 2016;25. 10.1002/ana.24936 28437840

[22] Elhai JD, de Francisco Carvalho L, Miguel FK, Palmieri PA, Primi R, Christopher Frueh B. Testing whether posttraumatic stress disorder and major depressive disorder are similar or unique constructs. J Anxiety Disord 2011;25:404–410. 21129914 10.1016/j.janxdis.2010.11.003

[23] Miller RG, Jackson CE, Kasarskis EJ, et al. Practice parameter update: the care of the patient with amyotrophic lateral sclerosis: multidisciplinary care, symptom management, and cognitive/behavioral impairment (an evidence-based review): report of the Quality Standards Subcommittee of the American Academy of Neurology. Neurology 2009;73:1227–1233. 19822873 10.1212/WNL.0b013e3181bc01a4 PMC2764728

[24] Woolley JD, Khan BK, Murthy NK, Miller BL, Rankin KP. The diagnostic challenge of psychiatric symptoms in neurodegenerative disease: rates of and risk factors for prior psychiatric diagnosis in patients with early neurodegenerative disease. J Clin Psychiatry 2011;72:126–133. 21382304 10.4088/JCP.10m06382oli PMC3076589

[25] McLaughlin RL, Schijven D, van Rheenen W, et al. Genetic correlation between amyotrophic lateral sclerosis and schizophrenia. Nat Commun 2017;8:14774. 28322246 10.1038/ncomms14774 PMC5364411

[26] Meng E, Yu S, Dou J, et al. Association between alcohol consumption and amyotrophic lateral sclerosis: a meta-analysis of five observational studies. Neurological Sci 2016;37:1203–1208. 10.1007/s10072-016-2575-0 27103621

[27] Huisman MH, Seelen M, van Doormaal PT, et al. Effect of presymptomatic body mass index and consumption of Fat and alcohol on amyotrophic lateral sclerosis. JAMA Neurol 2015;72:1155–1162. 26280944 10.1001/jamaneurol.2015.1584

[28] Ji J, Sundquist J, Sundquist K. Association of alcohol use disorders with amyotrophic lateral sclerosis: a Swedish national cohort study. Eur J Neurol 2016;23:270–275. 25641323 10.1111/ene.12667

[29] De Jong SW, Huisman MH, Sutedja NA, et al. Smoking, alcohol consumption, and the risk of amyotrophic lateral sclerosis: a population-based study. Am J Epidemiol 2012;176:233–239. 22791740 10.1093/aje/kws015

[30] D'Ovidio F, d'Errico A, Farina E, Calvo A, Costa G, Chio A. Amyotrophic lateral sclerosis incidence and previous prescriptions of drugs for the nervous system. Neuroepidemiology 2016;47:59–66. 27561959 10.1159/000448618

[31] Huang B, Dawson DA, Stinson FS, et al. Prevalence, correlates, and comorbidity of nonmedical prescription drug use and drug use disorders in the United States: results of the National Epidemiologic Survey on Alcohol and Related Conditions. J Clin Psychiatry 2006;67:1062–1073. 16889449 10.4088/jcp.v67n0708

[32] Kessler RC, Berglund P, Demler O, et al. The epidemiology of major depressive disorder: results from the National Comorbidity Survey Replication (NCS-R). JAMA 2003;289:3095–3105. 12813115 10.1001/jama.289.23.3095

[33] Furnari M, Epstein DH, Phillips KA, et al. Some of the people, some of the time: field evidence for associations and dissociations between stress and drug use. Psychopharmacology 2015;232:3529–3537. 26153066 10.1007/s00213-015-3998-7

[34] Tramonti F, Bongioanni P, Leotta R, Puppi I, Rossi B. Age, gender, kinship and caregiver burden in amyotrophic lateral sclerosis. Psychol Health Med 2015;20:41–46. 24588639 10.1080/13548506.2014.892627

[35] Gislason TB, Ostling S, Borjesson-Hanson A, et al. Effect of diagnostic criteria on prevalence of frontotemporal dementia in the elderly. Alzheimers Dement 2015;11:425–433. 24954370 10.1016/j.jalz.2014.03.002

