# Supplementary material for: Neurodegenerative and psychiatric diseases among families with amyotrophic lateral sclerosis
Source: Neurology. 2017 Aug 8;89(6):578–85. doi: 10.1212/WNL.0000000000004179 (PMC5562958; doi:10.1212/WNL.0000000000004179)
Supplement: Data Supplement [file supp_89_6_578__index.html]

Neurodegenerative and psychiatric diseases among families with amyotrophic lateral sclerosis — Data Supplement 

# Neurodegenerative and psychiatric diseases among families with amyotrophic lateral sclerosis

## Data Supplement

**Neurology® data supplements are not copyedited before publication. Published editorials and translations have been copyedited.  
 © 2017 American Academy of Neurology.  
  
 Files in this Data Supplement:**

- e-Tables - Microsoft Word file
