# Supplementary material for: Neurodegenerative and psychiatric diseases among families with amyotrophic lateral sclerosis
Source: Neurology. 2017 Aug 8;89(6):578–85. doi: 10.1212/WNL.0000000000004179 (PMC5562958; doi:10.1212/WNL.0000000000004179)
Supplement: Data Supplement [file supp_WNL.0000000000004179_e-tables.docx]

# Supplementary Table e-1

| International Classification of Diseases (ICD) codes used to identify amyotrophic lateral sclerosis (ALS), neurodegenerative diseases, and psychiatric disorders in the Swedish Patient Register | | | | | | | |
| --- | --- | --- | --- | --- | --- | --- | --- |
| **ICD-8** | **1969-1986** |  | **ICD-9** | **1987-1996** |  | **ICD-10** | **1997-2013** |
| **ALS** | | | | | | | |
| 348.00 | Amyotrophic lateral sclerosis |  | 335.C | Amyotrophic lateral sclerosis |  | G12.2 | Motor neuron disease |
| **Frontotemporal dementia** | | | | | | | |
| 290.11 | Pick’s disease |  | 331.B | Pick’s disease |  | F02.0 | Dementia in Pick’s disease |
|  |  |  |  |  |  | G31.0 | Pick’s disease |
| **Alzheimer's disease** | | | | | | | |
| 290.10 | Alzheimer's disease |  | 290.A^a^ | Senile dementia |  | F00 | Dementia in Alzheimer's disease |
|  |  |  | 290.B^a^ | Pre-senile dementia |  | G30 | Alzheimer's disease |
|  |  |  | 331.A | Alzheimer's disease |  |  |  |
| **Other or unspecific dementia** | | | | | | | |
| 290^b^ | Senile and pre-senile dementia |  | 290.A | Senile dementia |  | F01 | Vascular dementia |
| 293.0 | Psychosis with arteriosclerosis |  | 290.B | Pre-senile dementia |  | F02^c^ | Dementia in other diseases classified elsewhere |
| 293.1 | Psychosis with other cerebrovascular condition |  | 290.E | Vascular dementia |  | F03 | Unspecified dementia |
|  |  |  | 290.W | Other specified senile psychotic conditions |  | F05.1 | Delirium superimposed on dementia |
|  |  |  | 290.X | Unspecified senile psychotic condition |  | G31.1 | Senile degeneration of brain |
|  |  |  | 294.B | Dementia in conditions classified elsewhere |  | G31.8A^d^ | Lewy body disease |
|  |  |  | 331.C | Senile degeneration of brain |  |  |  |
|  |  |  | 331.X | Unspecified cerebral degeneration |  |  |  |
| **Parkinsonian disorders** | | | | | | | |
| 342.00 | Parkinson's disease |  | 332.A | Parkinson's disease |  | F02.3^d^ | Dementia in Parkinson's disease |
| 342.08 | Other defined parkinsonism |  | 333.A | Other degenerative diseases of the basal ganglia |  | G20 | Parkinson's disease |
| 342.09 | Unspecified parkinsonism |  |  |  |  | G21.4 | Vascular parkinsonism |
|  |  |  |  |  |  | G21.8 | Other defined secondary parkinsonism |
|  |  |  |  |  |  | G21.9 | Unspecified secondary parkinsonism |
|  |  |  |  |  |  | G23.1 | Progressive supranuclear ophthalmoplegia |
|  |  |  |  |  |  | G23.2 | Striatonigral degeneration |
|  |  |  |  |  |  | G23.9 | Unspecified degenerative disease of basal ganglia |
|  |  |  |  |  |  | G25.9 | Unspecified extrapyramidal and movement disorder |
|  |  |  |  |  |  | G31.8A^d^ | Lewy body disease |
| **Schizophrenia** | | | | | | | |
| 295 | Schizophrenia |  | 295 | Schizophrenia |  | F20 | Schizophrenia |
| **Bipolar disorder** | | | | | | | |
| 296.1 | Affective psychosis manic-depressive, manic type |  | 296.A | Unipolar-Manic affective psychosis |  | F30 | Manic episode |
|  |  |  |  |  |  |  |  |
| 296.3 | Affective psychosis manic-depressive, circular type |  | 296.C | Bipolar affective psychosis, manic phase |  | F31 | Bipolar disorder |
| 296.8 | Affective psychosis manic-depressive, other specified |  | 296.D | Bipolar affective psychosis, melancholic phase |  |  |  |
|  |  |  | 296.E | Bipolar affective psychosis, mild form |  |  |  |
|  |  |  | 296.W | Other specified affective psychosis |  |  |  |
| **Depression** | | | | | | | |
| 300.4 | Depressive neurosis |  | 300.E | Depressive neurosis |  | F32^e^ | Depressive episode |
|  |  |  | 311 | Depression not otherwise specified |  | F33^f^ | Recurrent depressive disorders |
|  |  |  |  |  |  | F34 | Chronic mood disorder |
|  |  |  |  |  |  | F38 | Other mood disorders |
|  |  |  |  |  |  | F39 | Unspecified mood disorder |
| **Neurotic disorders** | | | | | | | |
| 300.1 | Anxiety disorder |  | 300.A | Anxiety disorder |  | F40 | Phobic disorder |
| 300.2 | Phobic disorder |  | 300.B | Hysteria |  | F41 | Other anxiety disorders |
| 300.3 | Obsessive compulsive disorder |  | 300.C | Phobic disorder |  | F42 | Obsessive compulsive disorder |
| 300.5 | Neurasthenia |  | 300.D | Obsessive compulsive |  | F44 | Dissociative syndrome |
| 300.6 | Depersonalization disorder |  | 300.F | Neurasthenia |  | F45 | Somatoform disorder |
| 300.7 | Hypochondria |  | 300.G | Depersonalization disorder |  | F48 | Other neurotic disorders |
| 300.8 | Other specified neurotic disorder |  | 300.H | Hypochondria |  |  |  |
| 300.9 | Not otherwise specified |  | 300.W | Other specified neurotic disorder |  |  |  |
|  |  |  | 300.X | Unspecified |  |  |  |
| **Stress-related disorders** | | | | | | | |
| 307 | Transient situational disturbances |  | 308 | Acute reaction to stress |  | F43 | Adjustment disorder and reaction to severe stress |
|  |  |  | 309 | Adjustment disorder |  |  |  |
| **Alcohol abuse/dependence** | | | | | | | |
| 303 | Alcoholism |  | 303 | Alcoholism |  | F10^g^ | Mental and behavioral disorders caused by alcohol |
|  |  |  | 305.A | Non-dependent alcohol abuse |  |  |  |
| **Drug abuse/dependence** | | | | | | | |
| 304 | Drug addiction, abuse of drugs |  | 304 | Drug addiction |  | F11 | Mental and behavioral disorders due to use of opioids |
|  |  |  | 305.X | Abuse of drugs and medicines |  | F12 | Mental and behavioral disorders caused by cannabis |
|  |  |  |  |  |  | F13 | Mental and behavioral disorders due to use of sedatives and hypnotics |
|  |  |  |  |  |  | F14 | Mental and behavioral disorders due to use of cocaine |
|  |  |  |  |  |  | F15 | Mental and behavioral disorders due to use of other stimulants, including caffeine |
|  |  |  |  |  |  | F16 | Mental and behavioral disorders due to use of hallucinogens |
|  |  |  |  |  |  | F17 | Mental and behavioral disorders due to use of tobacco |
|  |  |  |  |  |  | F18 | Mental and behavioral disorders due to use of volatile solvents |
|  |  |  |  |  |  | F19 | Mental and behavioral disorders due to multiple drug use and use of other psychoactive substances |
| ^a^ If found as primary diagnosis | | | | | | | |
| ^b^ Except 290.10 (Alzheimer’s disease) and 290.11 (Pick’s disease) | | | | | | | |
| ^c^ Except F02.0 (Dementia in Pick’s disease) | | | | | | | |
| ^d^ ICD-10 codes considered both dementia and parkinsonian disorder diagnoses | | | | | | | |
| ^e^ Except F32.2 (depressive psychosis) | | | | | | | |
| ^f^ Except F33.3 (depressive psychosis) | | | | | | | |
| ^g^ Except F10.5 (psychotic state) | | | | | | | |

# Supplementary Table e-2

| Adjusted associations for age, sex, and county of birth among patients with amyotrophic lateral sclerosis (ALS) and their matched ALS-free controls show a higher-than-expected occurrence of neurodegenerative and psychiatric diseases after ALS diagnosis (hazard ratios); follow-up stopped at 69 years | |
| --- | --- |
|  | **After Index Date** |
|  |  |
|  | **HR (95%CI)*** |
| **Any neurodegenerative disease** | 8.01 (4.97-12.92) |
| **Other or unspecific dementia** | 13.66 (6.38-29.24) |
| *HR: hazard ratio; CI: confidence interval; Adjusted for age, sex, and county of birth | |

# Supplementary Table e-3

| Adjusted associations for age, sex, and county of birth among relatives of patients with amyotrophic lateral sclerosis (ALS) and relatives of their matched ALS-free controls do not show clear temporal pattern in the associations of ALS with neurodegenerative and psychiatric diseases | | | | | | | |
| --- | --- | --- | --- | --- | --- | --- | --- |
|  | **Prior to Index Date; OR (95% CI)^** | | |  | **After Index Date; HR (95% CI)^** | | |
|  | **≥ 6 years** | **2-5 years** | **0-1 year** |  | **0-1 year** | **2-5 years** | **≥ 6 years** |
| **Any neurodegenerative or psychiatric disease** |  |  |  |  |  |  |  |
| Parents | 0.88 (0.68-1.15) | 1.08 (0.79-1.48) | 1.3 (0.8-2.2) |  | 0.6 (0.2-1.7) | 1.21 (0.81-1.81) | 0.97 (0.69-1.35) |
| Siblings | 1.02 (0.90-1.16) | 1.01 (0.81-1.25) | 1.5 (1.0-2.1) |  | 1.0 (0.5-1.9) | 1.25 (0.95-1.65) | 1.29 (1.05-1.58) |
| Half-siblings | 0.8 (0.5-1.3) | 0.5 (0.2-1.3) | 0.2 (0.1-1.8) |  | n/a | 0.2 (0.1-1.5) | 2.3 (0.7-8.1) |
| Children | 1.07 (0.93-1.23) | 1.25 (1.06-1.47) | 0.9 (0.6-1.3) |  | 2.20 (1.32-3.66) | 1.07 (0.87-1.32) | 0.98 (0.82-1.16) |
| **Neurodegenerative diseases*** |  |  |  |  |  |  |  |
| Parents | 1.3 (0.7-2.3) | 1.0 (0.7-1.5) | 1.2 (0.6-2.2) |  | 0.9 (0.2-3.4) | 1.27 (0.77-2.10) | 0.91 (0.62-1.37) |
| Siblings | 1.6 (0.9-2.8) | 1.2 (0.7-1.9) | 1.8 (0.9-3.5) |  | 1.1 (0.1-8.3) | 1.5 (0.8-2.6) | 2.33 (1.57-3.41) |
| Half-siblings | n/a | n/a | n/a |  | n/a | n/a | n/a |
| Children | n/a | 3.3 (0.3-33.2) | n/a |  | n/a | n/a | 1.0 (0.2-5.0) |
| **Psychiatric disorders**** |  |  |  |  |  |  |  |
| Parents | 0.85 (0.64-1.13) | 1.1 (0.7-1.6) | 1.3 (0.6-2.9) |  | 0.4 (0.1-1.5) | 0.9 (0.4-1.7) | 0.9 (0.5-1.5) |
| Siblings | 1.01 (0.89-1.16) | 1.02 (0.81-1.29) | 1.4 (0.9-2.0) |  | 1.1 (0.5-2.2) | 1.18 (0.87-1.59) | 1.04 (0.82-1.32) |
| Half-siblings | 0.8 (0.5-1.3) | 0.5 (0.2-1.5) | 0.2 (0.1-1.9) |  | n/a | 0.2 (0.1-1.5) | 2.3 (0.7-8.1) |
| Children | 1.07 (0.93-1.23) | 1.24 (1.05-1.47) | 0.9 (0.6-1.3) |  | 2.19 (1.31-3.65) | 1.08 (0.87-1.33) | 0.98 (0.83-1.17) |
| * Including frontotemporal dementia, Alzheimer's disease, other or unspecific dementia, and Parkinson's disease | | | | | | | |
| ** Including schizophrenia, bipolar disorder, depression, neurotic disorder, stress-related disorders, alcohol abuse/dependence, and drug abuse/dependence | | | | | | | |
| ^ OR: odds ratio; HR: hazard ratio; CI: confidence interval; Adjusted for age and county of birth, and for age and county of birth of the proband | | | | | | | |

# Supplementary Table e-4

| Adjusted associations for age, sex, and county of birth among patients with amyotrophic lateral sclerosis (ALS) and their relatives, compared to ALS-free individuals, show a higher-than-expected occurrence of neurodegenerative and psychiatric diseases among ALS patients, of neurodegenerative diseases among siblings of ALS patients, and of psychiatric disorders among children of ALS patients, both before (odds ratios) and after (hazard ratios) ALS diagnosis; separate analysis for males | | | | | | | |
| --- | --- | --- | --- | --- | --- | --- | --- |
|  | **Prior to Index Date** | | |  | **After Index Date** | | |
| **Males** | **ALS patients/** | **ALS-free Controls/** | **OR (95% CI)^** |  | **ALS patients/** | **ALS-free Controls/** | **HR (95% CI)^** |
|  | **Relatives of** | **Relatives of** |  |  | **Relatives of** | **Relatives of** |  |
|  | **ALS Patients** | **ALS-free Controls** |  |  | **ALS Patients** | **ALS-free Controls** |  |
|  | N (%) | N (%) |  |  | N (%) | N (%) |  |
| **Any neurodegenerative or psychiatric disease** |  |  |  |  |  |  |  |
| Proband | 267 (12.22) | 2,041 (9.34) | 1.36 (1.18-1.56) |  | 130 (6.78) | 1,285 (6.49) | 2.77 (2.24-3.42) |
| Parents | 52 (7.09) | 554 (7.55) | 1.07 (0.75-1.52) |  | 63 (9.25) | 691 (10.18) | 0.97 (0.63-1.49) |
| Siblings | 226 (8.93) | 2.247 (8.98) | 1.06 (0.90-1.24) |  | 150 (6.50) | 1,363 (5.99) | 1.14 (0.90-1.44) |
| Half-siblings | 36 (11.11) | 368 (11.53) | 0.6 (0.3-1.3) |  | 23 (7.99) | 199 (7.04) | 1.0 (0.3-3.1) |
| Children | 211 (6.34) | 1,932 (5.64) | 1.16 (0.99-1.35) |  | 198 (6.36) | 1,637 (5.06) | 1.28 (1.07-1.54) |
| **Neurodegenerative diseases*** |  |  |  |  |  |  |  |
| Proband | 62 (2.84) | 238 (1.09) | 2.74 (2.05-3.67) |  | 51 (2.66) | 418 (2.11) | 3.46 (2.42-4.94) |
| Parents | 17 (2.32) | 208 (2.83) | 1.0 (0.5-1.8) |  | 45 (6.61) | 481 (7.09) | 1.1 (0.6-1.9) |
| Siblings | 24 (0.95) | 179 (0.72) | 1.5 (0.9-2.5) |  | 53 (2.30) | 316 (1.39) | 2.18 (1.36-3.49) |
| Half-siblings | 0 (0.00) | 7 (0.22) | n/a |  | 2 (0.69) | 13 (0.46) | n/a |
| Children | 1 (0.03) | 7 (0.02) | 1.4 (0.2-12.7) |  | 0 (0.00) | 18 (0.06) | n/a |
| **Psychiatric disorders**** |  |  |  |  |  |  |  |
| Proband | 216 (9.89) | 1,859 (8.51) | 1.18 (1.02-1.37) |  | 84 (4.38) | 945 (4.77) | 2.37 (1.84-3.06) |
| Parents | 38 (5.18) | 382 (5.20) | 1.1 (0.7-1.6) |  | 22 (3.23) | 257 (3.79) | 0.8 (0.4-1.6) |
| Siblings | 210 (8.29) | 2,128 (8.51) | 1.04 (0.88-1.22) |  | 109 (4.73) | 1,097 (4.82) | 1.00 (0.76-1.29) |
| Half-siblings | 36 (11.11) | 362 (11.34) | 0.6 (0.3-1.3) |  | 21 (7.29) | 189 (6.69) | 1.0 (0.3-3.1) |
| Children | 210 (6.31) | 1,927 (5.62) | 1.16 (0.99-1.35) |  | 198 (6.36) | 1,625 (5.03) | 1.30 (1.08-1.56) |
| * Including frontotemporal dementia, Alzheimer's disease, other or unspecific dementia, and Parkinson's disease | | | | | | | |
| ** Including schizophrenia, bipolar disorder, depression, neurotic disorder, stress-related disorders, alcohol abuse/dependence, and drug abuse/dependence | | | | | | | |
| ^ OR: odds ratio; HR: hazard ratio; CI: confidence interval; Adjusted for age and county of birth, and for age and county of birth of the proband in the analyses of relatives | | | | | | | |

# Supplementary Table e-5

| Adjusted associations for age, sex, and county of birth among patients with amyotrophic lateral sclerosis (ALS) and their relatives, compared to ALS-free individuals, show a higher-than-expected occurrence of neurodegenerative and psychiatric diseases among ALS patients, and of neurodegenerative diseases among siblings of ALS patients, both before (odds ratios) and after (hazard ratios) ALS diagnosis; separate analysis for females | | | | | | | |
| --- | --- | --- | --- | --- | --- | --- | --- |
|  | **Prior to Index Date** | | |  | **After Index Date** | | |
| **Females** | **ALS patients/** | **ALS-free Controls/** |  |  | **ALS patients/** | **ALS-free Controls/** |  |
|  | **Relatives of** | **Relatives of** | **OR (95% CI)^** |  | **Relatives of** | **Relatives of** | **HR (95% CI)^** |
|  | **ALS Patients** | **ALS-free Controls** |  |  | **ALS Patients** | **ALS-free Controls** |  |
|  | N (%) | N (%) |  |  | N (%) | N (%) |  |
| **Any neurodegenerative or psychiatric disease** |  |  |  |  |  |  |  |
| Proband | 212 (14.49) | 1,329 (9.08) | 1.70 (1.46-1.99) |  | 88 (7.03) | 853 (6.41) | 3.16 (2.40-4.15) |
| Parents | 114 (10.40) | 1,317 (10.86) | 1.02 (0.80-1.30) |  | 117 (11.91) | 1,383 (12.79) | 1.05 (0.80-1.39) |
| Siblings | 225 (9.27) | 2,170 (9.00) | 1.07 (0.91-1.25) |  | 155 (7.04) | 1,303 (5.94) | 1.29 (1.02-1.62) |
| Half-siblings | 22 (8.27) | 371 (11.98) | 0.5 (0.2-1.1) |  | 18 (6.72) | 217 (7.18) | 2.5 (0.7-8.8) |
| Children | 248 (7.75) | 2,275 (7.02) | 1.06 (0.91-1.22) |  | 220 (7.45) | 2,099 (6.96) | 1.00 (0.84-1.19) |
| **Neurodegenerative diseases*** |  |  |  |  |  |  |  |
| Proband | 57 (3.90) | 115 (0.79) | 5.26 (3.79-7.31) |  | 26 (2.08) | 245 (1.84) | 5.4 (3.1-9.2) |
| Parents | 56 (5.11) | 578 (4.77) | 1.18 (0.82-1.70) |  | 87 (8.86) | 975 (9.02) | 1.18 (0.84-1.66) |
| Siblings | 24 (0.99) | 194 (0.81) | 1.5 (0.9-2.4) |  | 40 (1.82) | 289 (1.32) | 1.6 (1.0-2.6) |
| Half-siblings | 3 (1.13) | 7 (0.23) | n/a |  | 1 (0.37) | 9 (0.30) | n/a |
| Children | 1 (0.03) | 7 (0.02) | 3.1 (0.3-35.3) |  | 3 (0.10) | 14 (0.05) | 6.0 (0.9-41.2) |
| **Psychiatric disorders**** |  |  |  |  |  |  |  |
| Proband | 166 (11.35) | 1,255 (8.58) | 1.37 (1.15-1.62) |  | 66 (5.28) | 650 (4.89) | 2.76 (2.03-3.76) |
| Parents | 71 (6.48) | 855 (7.05) | 0.94 (0.70-1.26) |  | 37 (3.77) | 514 (4.75) | 0.8 (0.5-1.3) |
| Siblings | 210 (8.65) | 2,023 (8.39) | 1.06 (0.90-1.25) |  | 116 (5.27) | 1,078 (4.92) | 1.17 (0.90-1.52) |
| Half-siblings | 19 (7.14) | 366 (11.81) | 0.6 (0.3-1.2) |  | 17 (6.34) | 211 (6.98) | 2.5 (0.7-8.8) |
| Children | 247 (7.71) | 2,272 (7.01) | 1.05 (0.91-1.22) |  | 218 (7.38) | 2,088 (6.93) | 0.99 (0.83-1.18) |
| * Including frontotemporal dementia, Alzheimer's disease, other or unspecific dementia, and Parkinson's disease | | | | | | | |
| ** Including schizophrenia, bipolar disorder, depression, neurotic disorder, stress-related disorders, alcohol abuse/dependence, and drug abuse/dependence | | | | | | | |
| ^ OR: odds ratio; HR: hazard ratio; CI: confidence interval; Adjusted for age and county of birth, and for age and county of birth of the proband in the analyses of relatives | | | | | | | |

# Supplementary Table e-6

| Adjusted associations for age, sex, and county of birth among patients with amyotrophic lateral sclerosis (ALS) and their relatives, compared to ALS-free individuals, show a higher-than-expected occurrence of neurodegenerative and psychiatric diseases among ALS patients, and of psychiatric disorders among children of ALS patients, both before (odds ratios) and after (hazard ratios) ALS diagnosis; separate analysis for individuals ≤55 years | | | | | | | |
| --- | --- | --- | --- | --- | --- | --- | --- |
|  | **Prior to Index Date** | | |  | **After Index Date** | | |
| **≤ 55 years at Index date** | **ALS patients/** | **ALS-free Controls/** | **OR (95% CI)^** |  | **ALS patients/** | **ALS-free Controls/** | **HR (95% CI)^** |
|  | **Relatives of** | **Relatives of** |  |  | **Relatives of** | **Relatives of** |  |
|  | **ALS Patients** | **ALS-free Controls** |  |  | **ALS Patients** | **ALS-free Controlss** |  |
|  | N (%) | N (%) |  |  | N (%) | N (%) |  |
| **Any neurodegenerative or psychiatric disease** |  |  |  |  |  |  |  |
| Proband | 96 (9.93) | 726 (7.51) | 1.36 (1.09-1.71) |  | 67 (7.69) | 636 (7.11) | 2.45 (1.82-3.30) |
| Parents | 8 (7.34) | 73 (7.14) | 0.9 (0.4-2.3) |  | 7 (6.93) | 80 (8.42) | 0.5 (0.2-1.6) |
| Siblings | 141 (7.44) | 1,426 (7.52) | 0.95 (0.77-1.16) |  | 139 (7.92) | 1,252 (7.14) | 1.18 (0.95-1.46) |
| Half-siblings | 37 (9.44) | 485 (11.47) | 0.5 (0.3-1.0) |  | 30 (8.45) | 334 (8.92) | 1.0 (0.4-2.1) |
| Children | 455 (7.02) | 4,137 (6.25) | 1.12 (1.01-1.25) |  | 418 (6.94) | 3,730 (6.01) | 1.11 (0.99-1.25) |
| **Neurodegenerative diseases*** |  |  |  |  |  |  |  |
| Proband | 19 (1.96) | 7 (0.07) | 27.1 (11.4-64.6) |  | 15 (1.72) | 48 (0.54) | 6.7 (2.8-15.9) |
| Parents | 0 (0.00) | 0 (0.00) | n/a |  | 1 (0.99) | 6 (0.63) | 3.3 (0.2-45.1) |
| Siblings | 2 (0.11) | 19 (0.10) | 0.9 (0.2-5.1) |  | 16 (0.91) | 108 (0.62) | 1.8 (0.9-3.5) |
| Half-siblings | 0 (0.00) | 4 (0.09) | n/a |  | 1 (0.28) | 9 (0.24) | n/a |
| Children | 1 (0.02) | 12 (0.02) | 1.1 (0.1-9.3) |  | 3 (0.05) | 32 (0.05) | 1.0 (0.3-3.5) |
| **Psychiatric disorders**** |  |  |  |  |  |  |  |
| Proband | 81 (8.38) | 722 (7.47) | 1.13 (0.89-1.44) |  | 55 (6.31) | 600 (6.71) | 2.25 (1.64-3.08) |
| Parents | 8 (7.34) | 73 (7.14) | 0.9 (0.4-2.3) |  | 6 (5.94) | 78 (8.21) | 0.4 (0.1-1.3) |
| Siblings | 139 (7.34) | 1,413 (7.45) | 0.94 (0.76-1.15) |  | 128 (7.30) | 1,175 (6.70) | 1.12 (0.90-1.40) |
| Half-siblings | 37 (9.44) | 483 (11.42) | 0.5 (0.3-1.0) |  | 29 (8.17) | 329 (8.79) | 1.0 (0.4-2.1) |
| Children | 454 (7.00) | 4,130 (6.24) | 1.12 (1.01-1.24) |  | 416 (6.90) | 3,707 (5.97) | 1.11 (0.99-1.25) |
| * Including frontotemporal dementia, Alzheimer's disease, other or unspecific dementia, and Parkinson's disease | | | | | | | |
| ** Including schizophrenia, bipolar disorder, depression, neurotic disorder, stress-related disorders, alcohol abuse/dependence, and drug abuse/dependence | | | | | | | |
| ^ OR: odds ratio; HR: hazard ratio; CI: confidence interval; Adjusted for age and county of birth, and for age and county of birth of the proband in the analyses of relatives | | | | | | | |

# Supplementary Table e-7

| Adjusted associations for age, sex, and county of birth among patients with amyotrophic lateral sclerosis (ALS) and their relatives, compared to ALS-free individuals, show a higher than expected occurrence of neurodegenerative and psychiatric diseases among ALS patients, and of neurodegenerative diseases among siblings of ALS patients, both before (odds ratios) and after (hazard ratios) ALS diagnosis; separate analyses for individuals ≥56 years | | | | | | | | |
| --- | --- | --- | --- | --- | --- | --- | --- | --- |
|  | **Prior to Index Date** | | |  | **After Index Date** | | | |
| **≥ 56 years at Index date** | **ALS patients/** | **ALS-free Controls/** | **OR (95% CI)^** |  | **ALS patients/** | **ALS-free Controls/** | | **HR (95% CI)^** |
|  | **Relatives of** | **Relatives of** |  |  | **Relatives of** | **Relatives of** | |  |
|  | **ALS Patients** | **ALS-free Controls** |  |  | **ALS Patients** | **ALS-free Controls** | |  |
|  | N (%) | N (%) |  |  | N (%) | N (%) | |  |
| **Any neurodegenerative or psychiatric disease** |  |  |  |  |  |  | |  |
| Proband | 383 (14.29) | 2,644 (9.86) | 1.53 (1.36-1.72) |  | 151 (6.57) | 1,502 (6.22) | | 3.15 (2.57-3.86) |
| Parents | 158 (9.19) | 1,798 (9.75) | 1.01 (0.82-1.23) |  | 173 (11.08) | 1,994 (11.98) | | 1.10 (0.88-1.3) |
| Siblings | 310 (10.11) | 2,991 (9.92) | 1.06 (0.93-1.21) |  | 166 (6.03) | 1,414 (5.21) | | 1.36 (1.10-1.68) |
| Half-siblings | 21 (10.61) | 254 (12.32) | 0.5 (0.2-1.7) |  | 9 (5.08) | 76 (4.21) | | n/a |
| Children | 4 (8.70) | 70 (13.73) | 1.0 (0.3-3.9) |  | 0 (0.00) | 6 (1.36) | | n/a |
| **Neurodegenerative diseases*** |  |  |  |  |  |  | |  |
| Proband | 100 (3.73) | 346 (1.29) | 3.05 (2.42-3.83) |  | 62 (2.70) | 615 (2.54) | | 3.71 (2.68-5.13) |
| Parents | 73 (4.24) | 786 (4.26) | 1.08 (0.80-1.46) |  | 131 (8.39) | 1,450 (8.71) | | 1.17 (0.91-1.50) |
| Siblings | 46 (1.50) | 354 (1.17) | 1.6 (1.1-2.3) |  | 77 (2.79) | 497 (1.83) | | 1.81 (1.29-2.53) |
| Half-siblings | 3 (1.52) | 10 (0.49) | n/a |  | 2 (1.13) | 12 (0.66) | | n/a |
| Children | 1 (2.17) | 2 (0.39) | n/a |  | 0 (0.00) | 0 (0.00) | | n/a |
| **Psychiatric disorders**** |  |  |  |  |  |  | |  |
| Proband | 301 (11.23) | 2,392 (8.92) | 1.29 (1.14-1.47) |  | 95 (4.13) | 995 (4.12) | | 2.71 (2.10-3.48) |
| Parents | 101 (5.87) | 1,164 (6.31) | 0.95 (0.75-1.21) |  | 53 (3.39) | 693 (4.16) | | 0.89 (0.62-1.28) |
| Siblings | 281 (9.17) | 2,738 (9.08) | 1.05 (0.91-1.21) |  | 97 (3.52) | 1,000 (3.68) | | 1.16 (0.89-1.50) |
| Half-siblings | 18 (9.09) | 245 (11.89) | 0.5 (0.2-1.7) |  | 7 (3.95) | 66 (3.65) | | n/a |
| Children | 3 (6.52) | 69 (13.53) | 0.8 (0.2-3.5) |  | 0 (0.00) | 6 (1.36) | | n/a |
| * Including frontotemporal dementia, Alzheimer's disease, other or unspecific dementia, and Parkinson's disease | | | | | | | | |
| ** Including schizophrenia, bipolar disorder, depression, neurotic disorder, stress-related disorders, alcohol abuse/dependence, and drug abuse/dependence | | | | | | | | |
| ^ OR: odds ratio; HR: hazard ratio; CI: confidence interval; Adjusted for age and county of birth, and for age and county of birth of the proband in the analyses of relatives | | | | | | | | |
| Supplementary Table e-8  \| Adjusted associations for age, sex, and county of birth among patients with familial amyotrophic lateral sclerosis (ALS) and their matched ALS-free controls show a higher-than-expected occurrence of neurodegenerative and psychiatric diseases among familial ALS patients, both before (odds ratios) and after(hazard ratios) ALS diagnosis \| \| \| \| \| \| \| \| \| --- \| --- \| --- \| --- \| --- \| --- \| --- \| --- \| \|  \| **Prior to Index Date** \| \| \|  \| **After Index Date** \| \| \| \|  \| **ALS** \| **ALS-free** \|  \|  \| **ALS** \| **ALS-free** \|  \| \|  \| **Patients** \| **Individuals** \| **OR (95% CI)^** \|  \| **Patients** \| **Individuals** \| **HR (95% CI)^** \| \|  \| N (%) \| N (%) \|  \|  \| N (%) \| N (%) \|  \| \| **Any neurodegenerative or psychiatric disease** \| 21 (12.14) \| 153 (8.84) \| 1.4 (0.9-2.4) \|  \| 10 (6.58) \| 113 (7.17) \| 3.1 (1.3-7.3) \| \| **Neurodegenerative diseases*** \| 5 (2.89) \| 9 (0.52) \| 5.9 (1.9-18.0) \|  \| 2 (1.32) \| 29 (1.84) \| 2.2 (0.2-20.1) \| \| **Psychiatric disorders**** \| 17 (9.83) \| 147 (8.50) \| 1.2 (0.7-2.0) \|  \| 8 (5.26) \| 92 (5.83) \| 3.1 (1.2-7.9) \| \| * Including frontotemporal dementia, Alzheimer's disease, other dementia, and Parkinson's disease \| \| \| \| \| \| \| \| \| ** Including schizophrenia, bipolar disorder, depression, neurotic disorder, stress-related disorders, alcohol abuse/dependence, and drug abuse/dependence \| \| \| \| \| \| \| \|  Supplementary Table e-9  \| Adjusted associations for age, sex, and county of birth among patients with amyotrophic lateral sclerosis (ALS) and their matched ALS-free controls show a higher-than-expected occurrence of neurodegenerative diseases among ALS patients, even after excluding frontotemporal dementia from the definition of neurodegenerative diseases \| \| \| \| \| \| \| \| \| --- \| --- \| --- \| --- \| --- \| --- \| --- \| --- \| \|  \| **Prior to Index Date; OR (95% CI)^** \| \| \|  \| **After Index Date; HR (95% CI)^** \| \| \| \|  \| **≥ 6 years** \| **2-5 years** \| **0-1 year** \|  \| **0-1 year** \| **2-5 years** \| **≥ 6 years** \| \| **Neurodegenerative diseases*** \| 1.60 (0.96-2.69) \| 2.18 (1.51-3.15) \| 8.80 (6.20-12.50) \|  \| 10.04 (6.14-16.44) \| 2.76 (1.64-5.63) \| 1.29 (0.58-2.86) \| \| * Including Alzheimer's disease, other dementia, and Parkinson's disease \| \| \| \| \| \| \| \| \| ^ Adjusted for age, sex, and county of birth \| \| \| \| \| \| \| \| | | | | | | | | |
|  | | | | | | |  |  |
|  | | | | | | |  |  |
